# Adaptation of the emerging pathogenic yeast *Candida auris* to high caspofungin concentrations correlates with cell wall changes

**DOI:** 10.1080/21505594.2021.1927609

**Published:** 2021-06-28

**Authors:** Violeta Lara-Aguilar, Cristina Rueda, Irene García-Barbazán, Sarai Varona, Sara Monzón, Pilar Jiménez, Isabel Cuesta, Ángel Zaballos, Óscar Zaragoza

**Affiliations:** a Mycology Reference Laboratory, National Centre for Microbiology, Instituto De Salud Carlos III, Madrid, Spain; b Bioinformatics Unit, Core Scientific and Technical Units, Instituto De Salud Carlos III, Madrid, Spain; c Genomics Unit, Core Scientific and Technical Units, Instituto De Salud Carlos III, Madrid, Spain

**Keywords:** *Candida auris*, trailing effect, paradoxical growth or Eagle effect, echinocandins, *FKS*, chitin, resistance, β-1,3-glucans

## Abstract

*Candida auris* has emerged as a fungal pathogen that causes nosocomial outbreaks worldwide. Diseases caused by this fungus are of concern, due to its reduced susceptibility to several antifungals. *C. auris* exhibits paradoxical growth (PG; defined as growth at high, but not intermediate antifungal concentrations) in the presence of caspofungin (CPF). We have characterized the cellular changes associated with adaptation to CPF. Using EUCAST AFST protocols, all *C. auris* isolates tested showed PG to CPF, although in some isolates it was more prominent. Most isolates also showed a trailing effect (TE) to micafungin and anidulafungin. We identified two *FKS* genes in *C. auris* that encode the echinocandins target, namely β-1,3-glucan synthase. *FKS1* contained the consensus hot-spot (HS) 1 and HS2 sequences. *FKS2* only contained the HS1 region which had a change (F635Y), that has been shown to confer resistance to echinocandins in *C. glabrata*. PG has been characterized in other species, mainly *C. albicans*, where high CPF concentrations induced an increase in chitin, cell volume and aggregation. In *C. auris* CPF only induced a slight accumulation of chitin, and none of the other phenomena. RNAseq experiments demonstrated that CPF induced the expression of genes encoding several GPI-anchored cell wall proteins, membrane proteins required for the stability of the cell wall, chitin synthase and mitogen-activated protein kinases (MAPKs) involved in cell integrity, such as *BCK2, HOG1* and *MKC1* (*SLT2*). Our work highlights some of the processes induced in *C. auris* to adapt to echinocandins.

## INTRODUCTION

In recent years, *Candida auris* has emerged as a multi-resistant pathogenic fungus associated with nosocomial environments, often in intensive care units (ICU) in several countries across five continents [1,2]. Diseases caused by *C. auris* pose a challenge for several reasons including: a) delayed detection due to the available phenotypic and biochemical methods and frequent confusion with other *Candida* species [3,4]; b) its ability to persist in a hospital environment as well as on the surface of medical instruments [2]; c) its ability to form biofilms and transmit from person to person [5,6]; d) its possession of virulence mechanisms and ability to evade the immune response or prevent the attack of neutrophils [7]; and e) its resistance to antifungal agents [6], as well as disinfectants used in daily practice [8].

*Candida auris* was first isolated in Japan in 2009 from a patient’s external ear canal [9]. Since then, this species has emerged almost simultaneously in other regions, including Asia, America, Africa and Europe [2,5,10–12]. The reason why *C. auris* has rapidly become a pathogen of concern in recent years is not known, but it has been suggested that some environmental changes (such as global warming) have favored that this species is becoming a part of our natural microbiota [13,14]. Genetic description of *C. auris* isolates from different geographical origins demonstrates that this species clusters into five different clades [11,12,15,16]. Diseases caused by *C. auris* have been associated with similar risk factors as for other *Candida* species infections, such as extremes of age, the presence of comorbidities, serious surgery, use of catheters and previous antifungal therapy [5,12,17]. However, unlike other *Candida* species, *C. auris* does not appear to effectively colonize the gastrointestinal tract, probably due to its poor growth under anaerobic conditions [11]. Different genomic studies have provided several factors that could be involved in its virulence mechanisms, such as production of lipases, oligopeptides transporters and siderophores [18]. Similarly to other opportunistic fungal pathogens, such as *C. albicans* or *C. glabrata* [19], *C. auris* has been shown to be virulent in mouse models.

Currently, there are four classes of antifungals available for treatment of candidiasis: azoles, polyenes (e.g. amphotericin B and nystatin), echinocandins (CPF caspofungin, MICA micafungin and ANIDU anidulafungin) and flucytosine. However, the treatment of diseases caused by *C. auris* is challenging because of different antifungal susceptibility profiles among clinical isolates and the ability to develop resistance to two or even three classes of antifungal drugs [12,20,21]. In particular, *C. auris* is fully resistant to fluconazole. The use of echinocandins as first-line therapies subject to a sensitivity testing has been recommended [17,22].

Echinocandins are semi-synthetic lipopeptides that present killing activity against *Candida* species, whose mechanism of action consists of noncompetitive inhibition of the Fks1p subunit of the enzyme β-1,3-glucan synthase [23]. Inhibition of this enzyme leads to cell lysis due to osmotic instability. Importantly, β-D-glucan is not present in mammalian cells, so side effects are minimal.

Although echinocandins are effective against most isolates of *Candida* species resistant to other antifungals, an increase in the number of *C. auris* strains with reduced susceptibility to one or more echinocandins has been observed (MIC ≥ 4 µg/mL for ANIDU and MICA; MIC ≥ 2 µg/mL for CPF) [24]. In fact, four hospitals in India have reported an alarming 37% resistance rate to CPF, determined by the microdilution method based on analysis of 102 isolates of *C. auris* [3].

The main mechanism by which *Candida* species have acquired resistance is through amino acid substitutions caused by mutations in two regions of the target gene *FKS1* and *FKS2*, which exhibit high recombination rates and are called hot-spots (HS1 and HS2) [25]. These mutations produce proteins with lower antifungal affinity [25]. So far, only three mutations have been described in *C. auris* isolates resistant to echinocandins, which take place in HS1 of *FKS1*; the substitution of amino acids S652Y and S639P [26,27], as well as the substitution in the same position S639F, equivalent to the mutation in position S645F that is associated with resistance to echinocandins in *C. albicans* [28].

In addition to the presence of mutations conferring resistance, recent studies indicate that *C. auris* has a peculiar sensitivity profile to echinocandins [29]. For a high number of isolates, echinocandins are unable to totally inhibit yeast growth. In particular, most isolates present paradoxical growth (PG, or Eagle effect) to CPF and exhibit “trailing” effect (TE, also known as residual growth or tolerance) to MICA and ANIDU *in vitro* during the antifungal susceptibility test (AFST) [30–38].

The “Eagle” or PG consists of the growth of yeasts at high concentrations of antifungal, although they remain susceptible to intermediate concentrations. This phenomenon has been observed in around 20–25% of isolates of some *Candida* species, such as *C. albicans* [30,32,39–42]. It has been associated with the activation of alternative pathways, morphological changes and alterations in the cell wall that compensate the defect of β-1,3-D-glucan, with the increase of chitin, causing an alteration in the immune response of the host [31,33,43].

TE is characterized by the absence of total inhibition of yeast growth with increasing concentrations of antifungal drugs, maintaining a residual growth at high drug concentrations. This phenomenon has been observed mainly with *Candida* species and azoles [44]. In this respect, strong trailing may result in the classification of strains as resistant, although clinical implications are not yet fully known.

In this work, we have studied the *in vitro* adaptation mechanisms of *C. auris* to echinocandins. For this purpose, we aimed to characterize the growth of isolates in the presence of caspofungin, micafungin and anidulafungin, and correlate it with possible changes in the cell wall. Finally, we also investigated the cellular changes induced by high caspofungin concentrations by RNAseq.

## MATERIALS AND METHODS

### Yeast strains and growth conditions

Seven clinical *C. auris* strains (Table 1) and three *C. albicans* isolates with different susceptibility profiles to echinocandins were used: CL-10449 (CalS, susceptible), CL-10432 (CalPG, strong PG in the presence of CPF) and CL-10272 (CalR, resistant strain that harbors the S645P mutation in HS1 of *FKS1*). As quality control strains, we included *C. krusei* ATCC 6258 and *C. parapsilosis* ATCC 22019.

**Table 1.** List of strains used in this work

Species	Strain	Geographical origin	Reference
*Candida auris*	CL-10838	Spain	MRL
	CL-10825	Spain	MRL
	CL-9998	Spain	MRL
	CL-10836	Spain	MRL
	CL-10958	Spain	MRL
	12272906	Colombia	MRL
	10263755	Colombia	MRL
	KCTC-17810	Korea	MRL
*Candida albicans*	CL-10432 (CalPG)	Spain	MRL
	CL-10449 (CalS)	Spain	MRL
	CL-10272 (CalR)	Spain	MRL
*Candida krusei*	ATCC 6258		ATTC
*Candida parapsilosis*	ATCC 22019		ATTC

MRL: Mycology Reference Laboratory; ATTC: American Type Culture Collection

Yeasts were cultured in Sabouraud liquid (Difco™, 0382–17) or Sabouraud agar (Oxoid, CM 0041) at 30°C.

### Antifungal susceptibility

*In vitro* antifungal susceptibility testing (AFST) was carried out according to EUCAST’s standardized yeast methodology using the plate microdilution method [45]. All plates included *C. parapsilosis* ATCC 22019 and *C. krusei* ATCC 6258 strains, as recommended by CLSI and EUCAST for quality.

RPMI-G medium was prepared with RPMI 1640 medium (Merck, Sigma-Aldrich, R-6504) buffered with MOPS (Merck, Sigma-Aldrich, M-1254) at pH 7 and supplemented with 2% glucose (Merck, Sigma-Aldrich, G-8270). Cell density was determined spectrophotometrically and the strains were inoculated at a final concentration of 1 to 5 × 10^5^ yeasts/mL. Antifungal concentration ranges were as follows: CPF (0.03 to 16 µg/mL) (Merck, Sigma-Aldrich, SML0425), MICA (0.004 to 2 µg/mL) (Molcan Corporation, 208538–73-2) and ANIDU (0.008 to 4 µg/mL) (Molcan Corporation, 166663–25-8). Plates were incubated at 35°C for 24 h in a humid atmosphere, and the optical density (OD) was measured at 530 nm after 24 h of incubation using an EZ Read 400 (Biochcrom, Cambridge, UK) microplates reading spectrophotometer. The minimum inhibitory concentration (MIC) was defined as the lowest antifungal concentration that caused a 50% growth inhibition compared to the control wells without antifungal. PG was defined when the yeasts were able to recover growth at high antifungal concentrations but inhibited at intermediate concentrations. TE was described when growth was inhibited by increasing concentrations of the antifungal, but this inhibition was not complete, and above a certain concentration, growth was not affected anymore.

### Growth curves

Growth curves were performed using microdilution plates with CPF concentrations between 0.03 to 16 µg/mL. Plates were inoculated with 1 to 5 × 10^5^ yeasts/mL and incubated at 35°C in a Multiskan FC spectrophotometer (ThermoFisher Scientific, Waltham, MA, USA). Optical densities (ODs) were measured at 540 nm each hour for 48 h. Results were analyzed using GraphPad Prism software, version 5.0. The lag period was established as the time needed to reach the basal OD and begin exponential growth. The doubling period was determined as the time in which the population doubled the OD, taking into account the logarithmic equation of the line obtained from the growth curves.

### DNA sequence analysis of FKS1 and FKS2 HS

To sequence the HS regions of the target genes *FKS1* and *FKS2* of *C. auris*, we amplified these genomic regions from CL-10836, CL-10838 and CL-10825 strains by PCR. Briefly, genomic DNA was extracted from individual colonies. Cells were disrupted with zirconia/silica beads (0.5 mm diameter) using FastPrep-24 (MP™, CA, USA) for 3 cycles, alternating 15 s shaking with 15 s on ice. DNA was obtained using phenol-chloroform and precipitated with 0.6x volumes of cold isopropanol. The pellet was washed with 70% ethanol and dissolved in 50 µL of distilled water with RNAse (40 µg/mL). Finally, DNA was puriﬁed using ChromaSPIN+ TE 200 columns (Clontech, 636082). The primers to amplify the HS1 and HS2 regions from *FKS1* and HS1 from *FKS2* were designed from the sequence with access number “MK059973.1” and “XM_029033096.1” available at GenBank, respectively (Table 2) and synthesized by Sigma-100 ENOSYS. To amplify a genomic region containing both HS1 and HS2 from *FKS1*, PCR was performed with primers FKS1-HS1-F and FKS1-HS2-R (0.5 µM) and 25 ng/mL of genomic DNA and employed the following thermal cycles: an initial step of 2 min at 98°C and 30 cycles of 98°C for 30 s, 72°C for 3 min with a ﬁnal extension step at 72°C for 7 min. A fragment of 2.601 bp was obtained, which was sequenced using the four primers designed for *FKS1* (see Table 2). To amplify the HS1 from *FKS2*, a fragment of 377 bp was obtained by PCR using the same genomic DNA and primers FKS2-HS1-F and FKS2-HS1-R. The PCR cycles were the same as described for *FKS1*, but with an amplification time of 1 min at 72°C.

**Table 2.** Sequence of the primers and probes used in the study

Gene	Primer or probe	Sense	Sequence (5´-3´)	Primer Size (bp)
*FKS1*	FKS1_HS1_F	Forward	TCTGCCATCTCGAAGTCTGC	20
	FKS1_HS1_R	Reverse	GCGAAATCAACACCTTTGGT	20
	FKS1_HS2_R	Reverse	ACCACCAACGGTCAAGTCTG	20
	FKS1_HS2_F	Forward	CCACGAATCCATTTTGTGTG	20
	FKS1_RT^1^_F	Forward	TGGTAGATTCATCGCCGACA	20
	FKS1_RT^1^_R	Reverse	GACCACCAGGATGAGACCTT	20
	FKS1 probe		6FAM-TGTACCGCTCATGTTAGCACCA-BHQ1	22
*FKS2*	FKS2_HS1_F	Forward	AAATGGAAGGGTTGCACTTG	20
	FKS2_HS1_R	Reverse	TCCAAGGCGTCCAGATAGAT	20
	FKS2_RT^1^_F	Forward	TACACCCAGCAACGCAATTT	20
	FKS2_RT^1^_R	Reverse	GCTGCGATTAAGCTGGGAAA	20
	FKS2 probe		6FAM–CATCTGCGTGAACATTGGCTT-BHQ1	21

^1^Used in Real Time PCR and Digital PCR

PCR products were treated with illustra™ ExoProStar™ (GE Healthcare Life Sciences, US77705). Puriﬁed PCR fragments were sequenced on both strands using 1 µM HS-F and HS-R primers and the BigDye™ Terminator v3.1 Cycle Sequencing Kit (Applied Biosystems, 4337457) to perform sequence reactions. Sequences were assembled and edited using SeqMan II and EditSeq Lasergene software programs (DNAstar, Madison, WI, USA).

### Estimation of chitin content and cellular volume

To evaluate changes in the cell wall of *C. auris* in response to different concentrations of CPF, chitin content was estimated by staining with Calcofluor White (CFW) (Merck, Sigma-Aldrich, F3543). Cells were inoculated in Sabouraud liquid medium and incubated at 30°C overnight with shaking (150 rpm). Cells were then washed with PBS and a suspension of 0.5 to 2.5 × 10^5^ cells/mL was prepared in RPMI-G. Five mL of these suspensions were incubated with different CPF concentrations (0, 0.06, 0.5 and 8 µg/mL) at 35°C with shaking for 24 h. Next, they were centrifuged at 2,500 rpm for 10 min and washed three times using 1 mL of PBS. Afterward, cells were fixed with 4% paraformaldehyde (Merck, Sigma-Aldrich, P6148) for 40 min at room temperature. The fixed cells were finally washed and suspended in 1 mL of PBS.

Cells were then incubated in PBS containing 1% bovine serum albumin (BSA, Merck, Sigma Aldrich, A4503) for 30 min at 37°C to avoid nonspecific binding. To detect chitin content on the cell wall, cells were incubated for 30 min with 10 μg/mL of CFW at 37°C in the dark. Samples were then washed with PBS and fluorescence was observed with a Leica SP5 confocal. Pictures (8 bits, 256 different gray intensities per pixel) were taken using LAS AF software (Leica Microsystems). The fluorescence intensity was estimated using Image J software (http://rsb.info.nih.gov/ij) and expressed as arbitrary units (scale from 0–255).

In addition, cellular volumes from at least 30 cells per sample were calculated by using the spheroid equation V = π/6 b^2^ × a, where “b” is the length and “a” is the width of the cell.

### Time-lapse recording and video processing

*Candida auris* and *C. albicans* cells were suspended at a final concentration of 0.5–2.5 × 10^5^ cells/mL in 96-well microtitre plates well containing CPF (0.5 and 8 µg/mL) in RPMI-G medium (see above). Plates were placed under a Leica SP5 confocal microscope which had a temperature-regulated chamber adjusted to 35°C. Images were taken with PMT-BF and excitation laser at 561 nm (AOTF at 8%), using a laser speed of 600 Hz. Pictures were taken every 5 min for 16 h using a 10x objective and 2x zoom. The .*lif* files were processed with ImageJ software and videos in .avi format were generated.

### Total RNA extraction in C. auris

A total of three samples were analyzed for each condition, each sample was from a different biological replicate obtained in different days. *Candida auris* CL-10836 was incubated in 100 mL of Sabouraud liquid medium overnight at 35°C with shaking (150 rpm). Cells were collected in exponential phase and washed with PBS. Cells were centrifuged at 15,000 g for 5 min and suspended in RPMI-G medium. A suspension of cells was inoculated in two equal fractions to a final concentration of 10^7^ cells/mL; one was used as a control without antifungal and the other was incubated with 8 µg/mL of CPF at 35°C with shaking (150 rpm). After 3 h, both aliquots were centrifuged at 12,500 rpm for 5 min and stored at −20°C.

RNA extraction was performed using Trizol (TRI Reagent®, Merck, Sigma-Aldrich, 93289) with some modifications. Cells were disrupted with zirconia/silica beads (0.5 mm diameter) using a FastPrep-24 (MP^TM^, CA, USA) for 4 cycles, alternating 40 s shaking with 1 min on ice. The extract was suspended in 50 µl of Ultra Pure Water and stored at −20°C. RNA concentration and quality was estimated using the Agilent 2100 Bioanalyzer. To eliminate DNA contamination from the RNA, all samples were treated with DNase using the DNA-free ™ DNA Removal Kit (Life Technologies S.A., Ambion, AM1906), according to the manufacturer’s instructions.

### Comparative gene expression of FKS1 and FKS2 using Real Time PCR

Extracted RNA was converted into complementary DNA (cDNA) using iScript^TM^ cDNA Synthesis Kit (Bio-Rad Laboratories, 1708891), according to the manufacturer´s instructions. Quantitative RT-qPCR was performed in a LightCycler 480 unit (Roche Diagnostics, Mannheim, Germany). Primer sequences for *FKS1* and *FKS2* are presented in Table 2. A final volume of 20 µl per reaction was prepared as follows: 1x SsoAdvanced Universal SYBR Green Supermix (Bio-Rad Laboratories, 1725270); 0.5 µM forward and reverse primer (FKS1 or FKS2) and 50 ng of cDNA. The RT-qPCR protocol had an initial step at 95°C for 30 s, followed by 40 cycles of amplification and quantification at 95°C for 30 s and 60°C for 1 min, with a single fluorescence measurement; and a final step at 95°C and continuous fluorescence measurement (melting curves). Real-time PCR efficiencies were estimated as previously described [46].

### Absolute quantification of FKS1 and FKS2 genes using Digital PCR

Primers designed for RT-qPCR were used in these experiments. Taqman probes targeting *C. auris FKS1* and *FKS2* genes were designed using Beacon Designer 7.0 software (Premier Biosoft, Palo Alto, CA) and synthesized by Merck, Sigma-Aldrich (Madrid, Spain). Sequences of primers and probes used in this study are detailed in Table 2. The number of *FKS1* and *FKS2* cDNA molecules was quantified using QuantStudio^TM^ 3D Digital PCR System (Applied Biosystems, A29154). Conditions for digital PCR were as follows: 1× QuantStudio 3D Digital PCR Mastermix v2 (Applied Biosystems, 4482710), 0.4 µM forward and reverse primer (*FKS1* or *FKS2*), 0.2 µM MB probe and 50 ng of cDNA (see above), in a final volume of 14.5 μl. Reactions were placed on a QuantStudioTM 3D Digital PCR 20 K Chip v2 (Applied Biosystems, A26317) automatically using QuantStudio^TM^ 3D Digital PCR Chip Loader (Applied Biosystems, CA, USA), and amplified following thermal cycling conditions recommended by manufacturer’s protocol. Applied Biosystems™ QuantStudio™ 3D AnalysisSuite™ Cloud Software was used for the analysis of data derived from the QuantStudio 3D Digital PCR instrument.

### Analysis of global gene expression using RNAseq

Libraries for RNAseq were prepared from 1 µg of total RNA (see Total RNA extraction in *C. auris*) using the TruSeq Stranded mRNA kit (Illumina, 20019792). Briefly, polyadenylated RNA was purified using oligodT beads, fragmented with divalent cations and converted to ds cDNA. Index adapters were then added and final libraries obtained after PCR. Quality and concentration of the libraries was estimated using Agilent 2100 Bioanalyzer and QuantiFluor® RNA System (Promega, E3310). A 4 nM pool containing equimolar fractions from each library was prepared, and 2 × 75 paired-end sequencing was performed in a NextSeq 550 Illumina platform using NextSeq 500/550 Mid Output Kit v2.5 (Illumina, 20024904).

The results were analyzed with a RNA-seq pipeline (https://github.com/BU-ISCIII/rnaseq-nf) written in Nextflow (https://www.nextflow.io/) based on the nf-core (https://nf-co.re/) previously written RNA-seq pipeline (https://github.com/nf-core/rnaseq). Fastq files containing raw reads were first analyzed for quality using fastQC v0.11.8 [http://www.bioinformatics.babraham.ac.uk/projects/fastqc/]. Raw reads were trimmed for low quality 3ʹ ends and adapter sequences removal using Trimmomatic v.0.38 [47]. The high-quality reads were then aligned against the *C. auris* reference genome GCA_002759435.2_Cand_auris_B8441_V2 using STAR v2.6.1d [48] and alignment quality control was performed using RseQC v3.0.0 22743226. Finally, transcriptome prediction and gene quantification were calculated using Subread’s featureCounts package v1.6.4 [49]. Original FastQ files were deposited at the Sequence Reads Archive (SRA, https://www.ncbi.nlm.nih.gov/sra) from the NCBI database (temporary Submission ID SUB8665675; BioProject ID PRJNA682422). The BioSample Accession numbers for the samples are SAMN16989163, SAMN16989164 and SAMN16989165 for the control samples (cells incubated in RPMI) and SAMN16989166, SAMN16989167 and SAMN16989168 for the three samples treated with CPF (8 µg/mL).

Many of the genes in *C. auris* are annotated as unknown proteins, but functional homologs can be found for the majority of these genes in the *C. albicans* and *S. cerevisiae* databases. For this reason, we performed an in-house annotation of the B8441 genome. The full list of all the ORFs from *C. auris* was compared in the *C. albicans* genome and a list of the homologs with the corresponding function was obtained. A similar approach was performed with the *S. cerevisiae* database. In this way, a list of all the *C. auris* genes with the corresponding homologs (when present) in *C. albicans* and *S. cerevisiae* was generated.

To characterize the functional categories of the identified CPF-regulated genes, two different types GO from the *Candida* genome database (candidagenome.org): GO Term finder (cellular function option) and GO Slim Mapper (component option) were used.

### Statistics

Differences in chitin content, cellular volume and changes in expression of *FKS* genes were assessed by t-Test using GraphPad software. ANOVA test with Bonferroni correction was used to determine differences in the number of copies of *FKS* genes (digital PCR). To identify differentially expressed genes (DEGs), differential expression analysis was carried out using DESeq2 R/Bioconductor package v1.18.1 [50]. DESeq2 was also used for normalization and results visualization.

## RESULTS

### *Susceptibility profile of* C. auris *strains to echinocandins*

**Figure 1.**
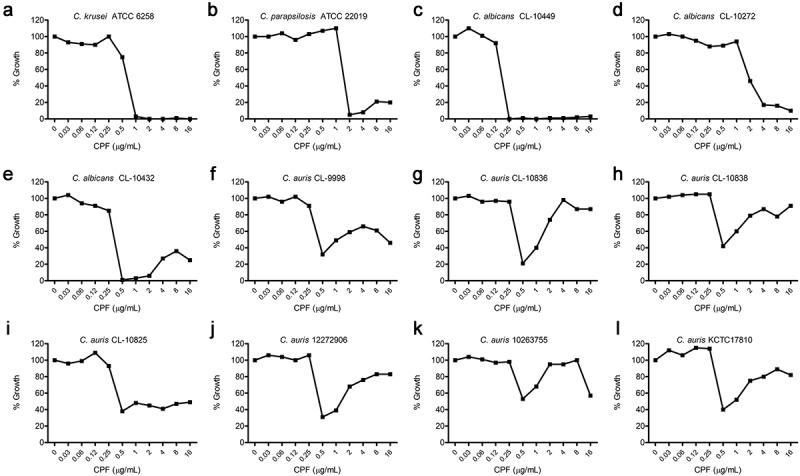
Characterization of *Candida* growth in the presence of CPF (0.03–16 µg/mL). *In vitro* susceptibility profile for the seven isolates of *C. auris*. The growth percentage represents the percentage of OD measured at 530 nm

The susceptibility profile of seven *C. auris* clinical isolates from different origins was compared with three *C. albicans* isolates that exhibited different susceptibility profiles to echinocandins (susceptible, resistant and paradoxical growth). All *C. auris* isolates exhibited similar PG in the presence of CPF (Figure 1). The CL-10836 strain showed the highest PG (Figure 1g), with an increase in growth of 77% with respect to the MIC. *C. albicans* strain CL-10432 also showed PG growth, being inhibition at intermediate concentrations practically total (Figure 1e). In contrast, growth reduction for all *C. auris* did not exceed 80% than that of the control growth (figure 1f to L). This growth capacity at increasing antifungal concentrations was different from the profile observed in a CaS (Figure 1c, CL-10449) or CaR strain (Figure 1d, CL-10272).

Micafungin and anidulafungin inhibited growth of *C. auris* (Supplemental Figures 1 and 2). However, most isolates showed residual growth (or trailing) at increasing antifungal concentrations (Supplemental Figures 2 and 3). This trailing was not present for any of the *C. albicans* nor the quality control strains (*C. krusei* and *C. parapsilosis*, Supplemental Figures 2 and 3). Therefore, these results indicated that *C. auris* has a special adaptation to echinocandins and that this adaptation was different from that observed in resistant strains of *C. albicans*.

**Figure 2.**
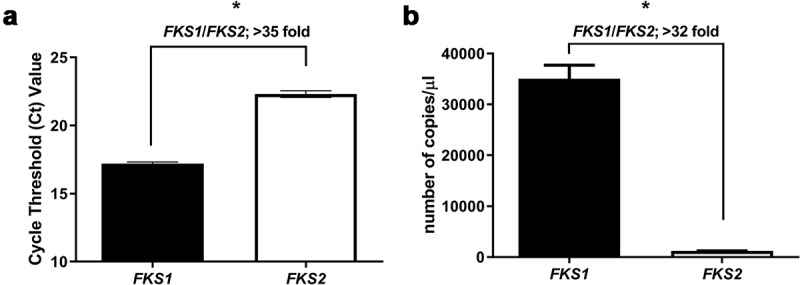
Expression of *FKS* genes in *C. auris* CL-10836 control cells. Cycle threshold value (Ct) of *FKS1* and *FKS2* genes determined by RT-qPCR (a); Number of cDNA copies per uL of *FKS1* gene (black bars) and *FKS2* gene (white bars) in control cells without antifungal (control) (b). * *p*< 0.001; statistical analysis was performed using Student’s t-test. The data are presented as the mean plus SD of 3 independent experiments

### Growth curves

To characterize changes in *C. auris* cell growth in response to CPF, growth curves with increasing concentrations of CPF were established. Test strains of *C. albicans* were CalR CL-10272 and CalPG CL-10432. At low CPF concentrations (0.06 µg/mL) both species showed a short lag period of 5 h, as well as an average doubling time similar to the growth control (around 6.5 h for *C. auris* and 4.9 h for *C. albicans*) (Table 3). However, at inhibitory concentrations (0.5 µg/mL) an increase in the lag period and duplication time was observed for both species. *Candida auris* grew slowly, with periods of lag and average doubling times that were 2 and 4.5 times greater than the growth control, respectively (Table 3). However, CalPG (CL10432) was the most affected strain, exhibiting a lag period 5.2 times longer and a doubling time 7.9 times longer than that the growth control (Table 3). No growth effects were found for the CalR strain with 0.5 µg/mL of CPF.

**Table 3.** Analysis of growth curves of *C. auris* and *C. albicans* in the presence of CPF. Lag period and doubling time of isolates of *C. auris, C. albicans* strain CL-10432 (CalPG) and *C. albicans* strain CL-10272 (CalR) in different concentrations of CPF (µg/mL)

Species	Strain	Lag period (h)					Doubling time (h)			
		CPF (µg/mL)				CPF (µg/mL)				
		0	0.06	0.5	8		0	0.06	0.5	8
*C. auris*	12272906	5	5	14	7		6.8	6.8	43.3	13.3
	10263755	5	5	13	8		6.6	6.2	34	14.2
	KCTC-17810	5	5	12	10		6.4	5.6	24.8	15.1
	CL-10838	5	5	7	6		6.5	5.7	42.5	21.7
	CL-10825	5	5	8	7		7.6	8.4	23	17.5
	CL-9998	5	5	8	7		6.1	6.7	22.1	13.8
	CL-10836	5	5	10	7		6.3	6.1	16.3	7.5
	**Average**	**5**	**5**	**10**	**7**		**6.5**	**6.6**	**29.6**	**14.8**
	**SD**	**0**	**0**	**2.5**	**1.1**		**0.5**	**0.9**	**9.3**	**3.8**
*C. albicans*	CL-10432 (CalPG)	5	5	26	8		4.9	4.9	38.7	11.4
	CL-10272 (CalR)	4	4	4	10		8	8.3	9.7	52.1

SD, standard deviation

Finally, at high CPF concentrations (8 µg/mL), growth of *C. auris* isolates and CalPG were comparable to the intermediate concentration. The average doubling period for *C. auris* isolates was around 2.3 times greater than that of the growth control, analogous to that observed for *C. albicans*. The lag period was 1.4 and 1.6 times longer than that for the growth control of *C. auris* and *C. albicans*, respectively (Table 3). However, this delay in the lag period did not affect the final growth of *C. auris* cells, as the yeasts achieved a final OD similar to that obtained with the control yeasts not exposed to CPF. As expected, growth of the CalR strain CL-10272 was markedly inhibited with high CPF concentrations (Table 3).

### *Sequencing of the hot spots of the* C. auris FKS *genes*

We next studied whether the adaptation of *C. auris* to echinocandins correlated with changes in the sequence of the target enzyme, β-1,3-glucan synthase. Firstly, a sequence analysis was performed on the genome of *C. auris*, where we identified two *FKS* genes. When the coding protein sequence was analyzed, the Fks1 enzyme gene had sequences of HS1 and HS2 regions. For the gene for the Fks2 enzyme, the HS1 region was detected, but not the HS2 region. For this reason, we designed primers to sequence the HS1 and HS2 regions of *FKS1* and HS1 of *FKS2*.

The three isolates analyzed (CL-10836, CL-10838 and CL-10825) showed the WT sequence of the reference genomes deposited in the databases. In all cases, the HS1 and HS2 regions from Fks1 had the sequence present in *Candida* susceptible species. Interestingly, in the HS1 from Fks2, we found that *C. auris* exhibited two changes compared to other susceptible species, namely substitutions of F635Y and R641K (Table 4). These changes were found in all reference genomes available in GenBank databases, so we assumed that this was the WT sequence from this species. It is noteworthy that the F635Y substitution has previously been shown to confer resistance to echinocandins in *C. glabrata* [51].

**Table 4.** Sequencing of *FKS1* hot spot 1 (HS1) and 2 (HS2) and *FKS2* HS1 from *C. auris* isolates. The amino acids highlighted correspond to the differences in HS1 between *FKS1* and *FKS2.*

Hot spot FKS	Nucleotide Sequence	Amino acid sequence
*FKS1* HS1	TTCTTCTTGACTTTGTCCTTGAGAGATCCT	^634^F**F**LTLSL**R**DP^643^
*FKS1* HS2	GACTGGATTAGACGTTATACCTTGTCC	^1350^DWIRRYTLS^1358^
*FKS2* HS1	TTCTATCTTACTCTCTCTTTGAAAGATCCT	^569^F**Y**LTLSL**K**DP^578^

### *Expression of* FKS *genes in* C. auris

Since HS1 from Fks2 showed a sequence that could potentially confer resistance to echinocandins, it could be argued that this was the reason of the adaptation of *C. auris* to these antifungals. However, the susceptibility profile observed in *C. auris* differed to the one observed in other resistant isolates from *Candida* species, such as *C. albicans* or *C. glabrata* with mutations in the HS. In these cases, changes in the HS regions result in reduced susceptibility, but not PG or TE (Figure 1). For this reason, the expression of both *FKS* genes was measured to determine the contribution of Fks2 in the adaptation to echinocandins.

RNA was isolated from control cells without antifungal, and gene expression of the *C. auris FKS* genes was studied by RT-qPCR and digital PCR. Real-time PCR efficiencies for both genes were >1.95 (data not shown). Using both techniques, RT-qPCR and digital PCR, we found that in *C. auris, FKS1* is more expressed than *FKS2*. When we compared the relative expression of both genes by RT-qPCR, *FKS1* was around 35-fold more expressed than *FKS2* (Figure 2a). Similar findings were observed by digital PCR, where the number of cDNA copies of *FKS1* was 32-fold higher than those of *FKS2* (Figure 2b).

### Cell wall chitin content in response to CPF

*Candida albicans* induces several changes to compensate the inhibition of the synthesis of β-1,3-glucans during CPF treatment, which include an increase of chitin in the cell wall [33,52]. To investigate whether adaptation to CPF in *C. auris* was associated with similar changes, the *C. auris* strains CL-10836 and CalPG CL-10432 were studied as they exhibit strong PG.

**Figure 3.**
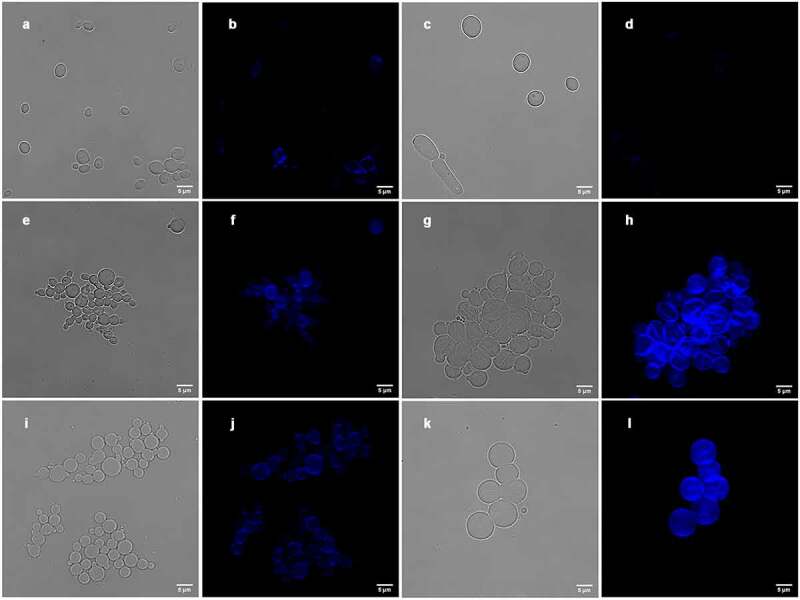
Changes in the wall of *C. auris* and *C. albicans* during PG. The images represent *C. auris* cells CL-10836 (a, b, e, f, i and j) and *C. albicans* CL-10432 (b, c, g, h, k and l) in different treatments: control cells (a–d), cells incubated with 0.5 µg/mL of CPF (e-h) and cells incubated with 8 µg/mL of CPF (i–l). Chitin was labeled with calcofluor and fluorescence was observed in a SP5 confocal microscope as described in M&M. The scale at the bottom right of the images represents 5 µm

**Figure 4.**
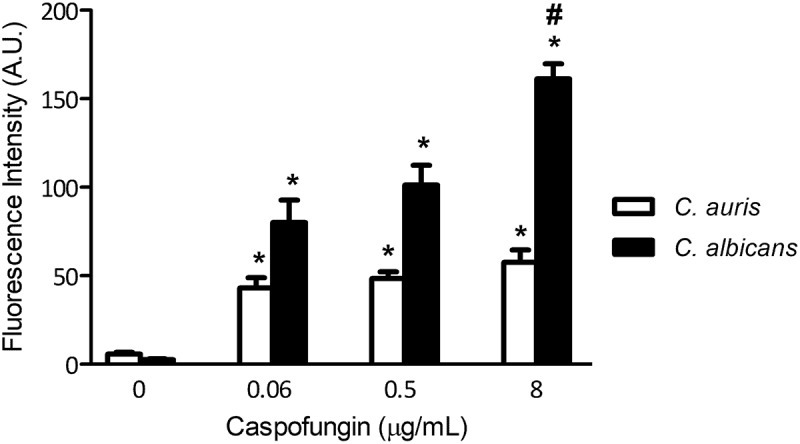
Quantification of the chitin content in the cell wall of *C. auris* CL-10836 and *C. albicans* CL-10432. The results are expressed in arbitrary intensity units. Statistical differences are shown in comparison with control cells (*) and between CPF concentrations of the same strain (#) (p<0.05). The fluorescence of around 30 cells was measured for each condition. The experiment was performed three times on different days, obtaining consistent results, and the results of a representative experiment are shown

To estimate the chitin content, we stained the cells with CFW and quantified chitin levels by fluorescence intensity. In the absence of CFW, the fluorescence signal in *C. auris* appeared to be higher than for *C. albicans*, although this difference was not statistically different (Figures 3a, b, c and d; Figure 4). Of note was that the chitin content significantly increased, not only at elevated antifungal concentrations, but also at concentrations below the MIC for both species (at 0.06 µg/mL, around 8-fold increase in *C. auris*, and around 30-fold increase in *C. albicans*, compared to the non-treated cells, Figures 3e-l and 4). The increase in chitin levels in response to increasing concentrations of CPF was more pronounced in *C. albicans*, especially between the inhibitory concentration of 0.5 µg/mL and 8 µg/mL (around 30-fold and 60-fold increase at 0.5 and 8 µg/mL, respectively, Figures 3 and 4). Surprisingly, even though *C. auris* subinhibitory CPF concentrations (0.06 µg/mL) induced chitin synthesis, higher concentrations of CPF did not result in any further increase in chitin content (around 9 and 10-fold increase at 0.5 and 8 µg/mL, respectively, Figure 4). These results suggest that survival of this yeast at elevated CPF concentrations was not as dependent on chitin synthesis as for *C. albicans*.

**Figure 5.**
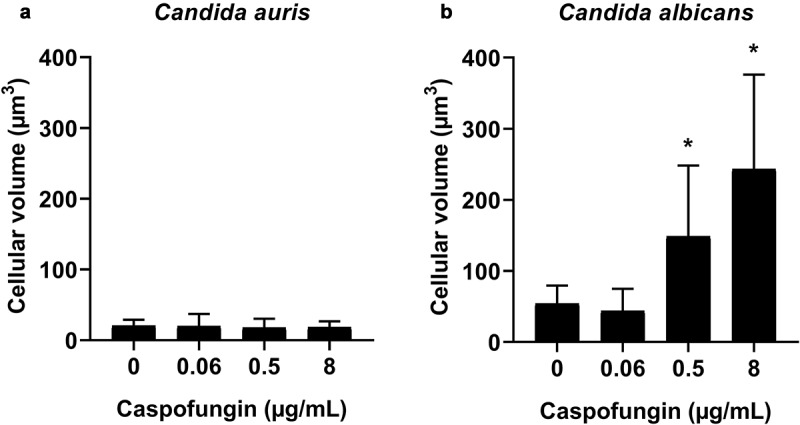
Distribution of cell volumes after incubation with different concentrations of CPF in *C. auris* CL-10836 (a) and *C. albicans* CL-10432 (b). The cellular volume of around 30 cells was measured for each condition and the average value and standard deviation are plotted as bar graph. The experiment was performed three times on different days, obtaining consistent results, and the results of a representative experiment are shown. Asterisks denote statistical difference between the sample and the control non-treated cells (p<0.05, Kruskal–Wallis test)

In a previous study, we observed that *C. albicans* cells increase their cellular volume in response to intermediate and high CPF concentrations [33]. Therefore, we investigated if the morphology of *C. auris* was affected by CPF. *Candida auris* had a cellular volume of around 20 µm^3^ in basal conditions. After CPF exposure, the cells became more spherical (Figure 3e and i), but did not have a significantly higher volume (Figure 5a). In comparison, *C. albicans* cells exhibited a higher volume in basal conditions, around 64 µm^3^. Increasing CPF concentrations resulted in the appearance of a new population of larger spherical cells, reaching cellular volumes of up to 530 µm^3^ at 8 µg/mL of CPF (Figures 3 and Figures 5b).

### *Real time visualization of the effect of CPF on* C. auris *morphology*

Adaptation of *C. albicans* to high CPF concentrations has been associated with morphological changes typical of a stress response, including increased cell volume and absence of the development of hyphae [33]. Therefore, studies were undertaken in real time to determine if the adaptation of *C. auris* resulted in similar changes. For this purpose, video analysis was undertaken encompassing the period of growth of CaPG CL-10432 and *C. auris* CL-10836 at different CPF concentrations.

**Figure 6.**
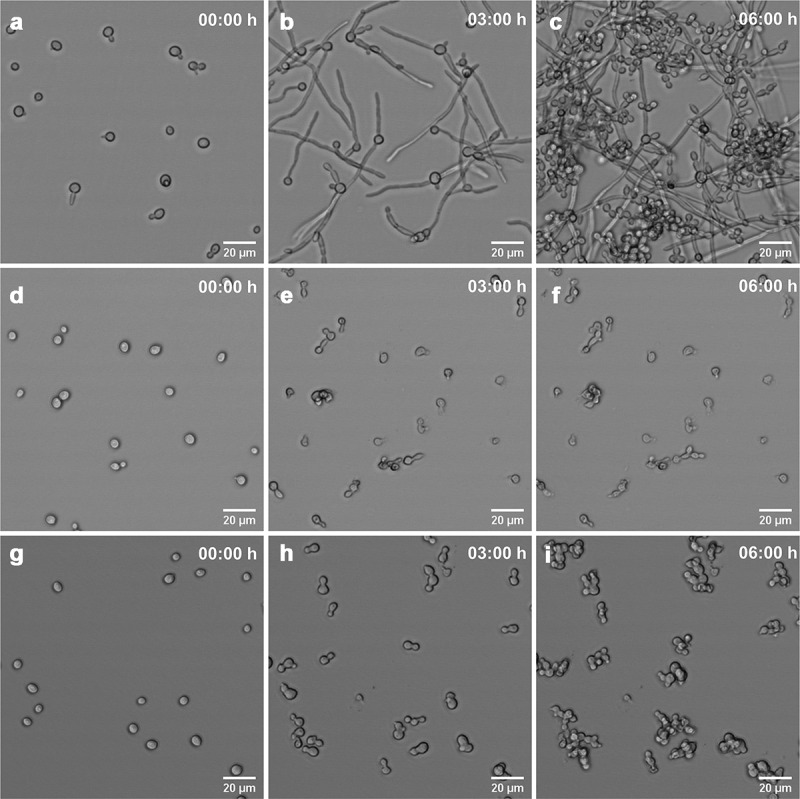
Changes in morphology and cell growth of *C. albicans* CL-10432. *In vivo* images show untreated cells (a-c), cells incubated with 0.5 µg/mL CPF (d-f) and cells incubated in the presence of 8 µg/mL of CPF (g-h) at initial time (a, d and g), 3 h (b, e and h) and 6 h (c, f and i)

**Figure 7.**
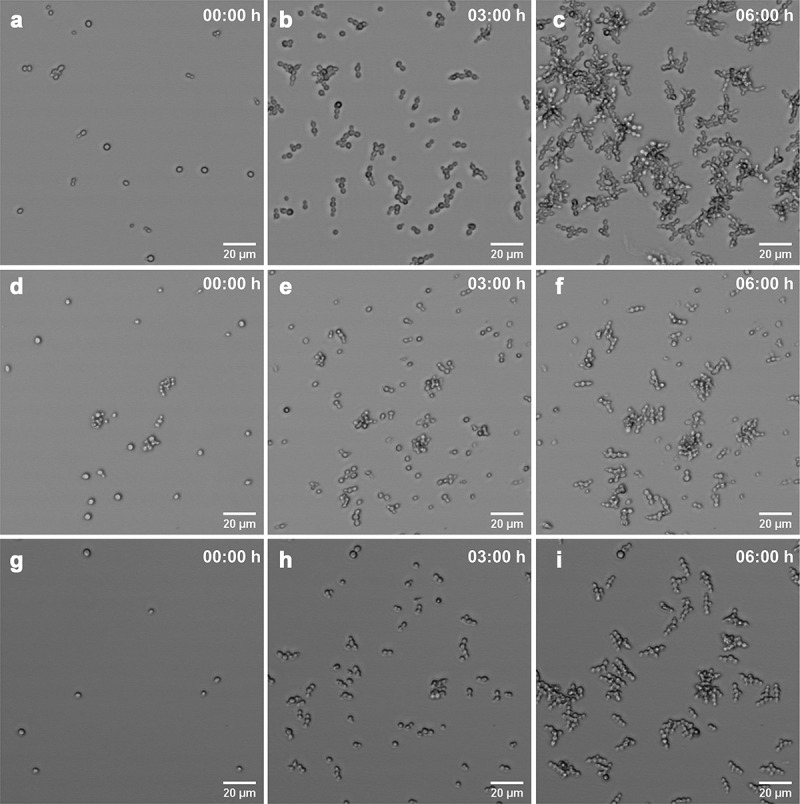
Changes in the morphology and cell growth of *C. auris* CL-10836. *In vivo* images show untreated cells (a-c), cells incubated with 0.5 µg/mL of CPF (d-f) and cells incubated in the presence of 8 µg/mL of CPF (g-i) at initial time (A, D and G), 3 h (B, E and H) and 6 h (C, F and I)

In the absence of CPF, CalPG rapidly formed filaments (Figure 6 a-c), which was not evident with *C. auris* (Figure 7 a-c). In agreement with the growth curves, intermediate CPF concentrations caused a marked growth inhibition which was much more pronounced for *C. albicans*, whose cells had an irregular shape, typical of exploded cells (supplemental video 1 and 2, and Figure 6 D-F and 7 D-F). At high CPF concentrations (8 µg/mL), whilst CalPG grew, cells did not form hyphae and exhibited a swollen and aggregated phenotype (Figure 6 D-I). In contrast, CPF did not cause any marked effect on *C. auris* growth or morphology at high CPF concentrations (Figure 7 G-I). These results suggested that the PG of *C. auris* was less affected at elevated CPF concentrations compared to *C.albicans*.

### Candida auris *gene expression in the presence of CPF*

To further investigate the cellular response of *C. auris* CL-10836 to CPF, total gene expression was quantified. RNA from control and CPF treated cells (8 µg/mL) were isolated and subjected to RNAseq using the Illumina platform.

**Figure 8.**
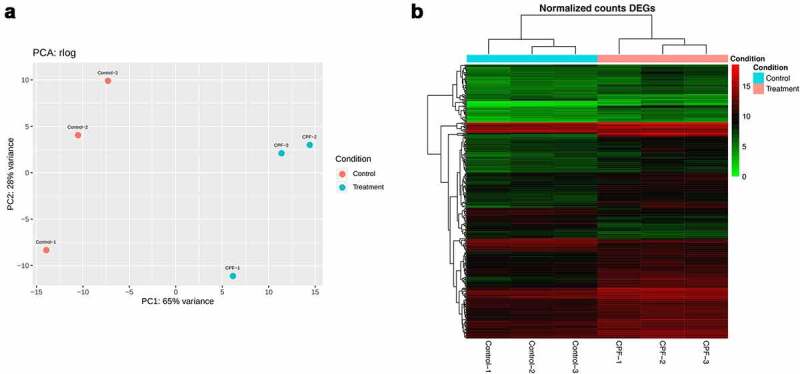
Results of the differential expression analysis. PCA plot of the top 500 DEGs. The PCA scores plot for PC1 and PC2 are shown with points colored by condition (Treatment or Control). The percentage of the total variance explained by each principal component is shown in brackets on each axis (a) and Heatmap plot of the 278 significantly over-expressed genes in CPF treated cells. The relationship between the color and the normalized expression values are indicated in the side bar. Each row represents one of the genes in the list (b)

Transcriptome analysis by RNA-Seq was used to compare untreated and treated cells. As shown in the Principal Component Analysis (PCA) plot in Figure 8a, treated samples and non-treated samples grouped separately based on the top 500 differentially expressed genes (DEGs). A total of 278 genes were overexpressed in the presence of CPF (>2 fold, p value<0.05, supplemental table 1). The normalized expression patterns of these 278 genes among the samples are illustrated in Figure 8b, where a clusterization of the treated cells versus untreated cells was observed. Examination of the function of the corresponding homologs in *C. albicans* and *S. cerevisiae* revealed that a significant proportion of genes encoded proteins related to cell wall. These included structural proteins of cell wall, membrane proteins involved in cell wall stability, enzymes required for the synthesis of the cell wall polysaccharides and MAPK required for cell integrity, cell wall rearrangements and stress response. The three most overexpressed genes after treatment with CPF encoded two GPI-anchored cell wall proteins homologous to *C. albicans*, Gpa31p and Gpa30p, and another cell wall structural mannoprotein homologous to *S. cerevisiae* Cwp1p. Interestingly, several overexpressed genes were involved in chitin synthesis, which is in agreement with the accumulation of chitin observed by fluorescence after CPF treatment. These included several genes for chitin synthases and enzymes for glucosamine metabolism, which are involved in the synthesis of UDP-N-acetyl glucosamine (precursor of chitin). It was also found that genes encoding for enzymes required for β-1,6-glucan were induced. Furthermore, CPF increased the expression of the Fks2-encoding gene by approximately 2-fold compared with control cells, which suggests a mechanism to compensate the inhibition of β-1,3 glucan caused by CPF. No significant differences in expression of the *FKS1* were found. It was also noteworthy to find that three of the genes that encoded the main MAPK involved in cell wall integrity (Bck1, Hog1 and Mck1, known as Slt2 in *S. cerevisiae*) were overexpressed in the presence of CPF.

To get a general view of the type of genes that exhibited increased expression, we performed a Gene Onthology Term Analysis. From this, a significant enrichment of genes belonging to the following functions was noted: 1) cell wall organization, structure, regulation and biosynthesis, 2) aminosugar, glucosamine and chitin synthesis and 3) RNA Polymerase II related (supplemental table 2). These results confirmed that gene expression changes in response to CPF were related to the cell wall structure and organization. Most of the genes described above were categorized in these functions by the GO algorithm. However, there were some genes, such *PGA30* and *PGA31*, that were not found among this list. For this reason, another GO analysis (GO Slim Mapper) was performed with classification of genes according to their cellular component (cell wall, cytoplasm, nucleus, etc.). Each gene family was manually identified that were related to cell wall and chitin synthesis. A list of 54 genes that were components of the cell wall or were related to this structure were identified (supplemental table 3).

In addition, CPF repressed expression of other genes encoding cell wall proteins, such as Iff4 and Rbt1 (involved in adhesion), Pga7, Pga45 and Scw11 (which presents homology to glucanases). Genes encoding chitinases were also significantly repressed (around 2–3 fold) by CPF. One of the most intriguing features was the finding that the most repressed genes by CPF encoded proteins were involved in zinc and iron uptake. To get a non-biased idea of the main families of genes that were repressed by CPF, a further GO Term analysis was performed and this revealed that amongst this set of genes, the main repressed functions were alcohol and ergosterol biosynthesis and metal ion homeostasis (transport into cell, supplemental table 4).

## DISCUSSION

*Candida auris* is a multi-drug resistant yeast and an emerging pathogen worldwide where it is responsible for invasive nosocomial infections that are often associated with high morbidity and mortality [53]. Although use of echinocandins is now recommended for treatment of *C. auris* infections, an increase in isolates with elevated MICs against this class of antifungals is being observed [54]. This microorganism has a particular ability to persist in hospital or medical material and has the ability to form biofilms, where the concentration of antifungal is higher than that of physiological fluids [53,55]. Therefore, the possibility of yeasts surviving treatment raises an additional concern that should be considered in cases of therapeutic failure.

The results obtained in this study indicated that echinocandins had no fungicidal activity against some *C. auris* isolates, where yeast growth was evident at concentrations equal to or higher than the MIC. Specifically, two phenomena have been detected *in vitro* using EUCAST protocol, PG at high CPF concentrations and a TE with MICA and ANIDU. To date, the latter is more frequent among other antifungals, such as azoles [36].

Our work also suggests that the MIC values of CPF, MICA and ANIDU against *C. auris* should be interpreted with care as they do not completely inhibit the growth of these isolates. In fact, in some studies they may be considered resistant after 48 h of treatment [29]. Therefore, this work may contribute to establishing criteria that help define whether *C. auris* strains with this type of adaptation should be considered *in vitro* as susceptible, intermediate or resistant to echinocandins. As described in [16], the isolates from the geographical origins used in this study (South Korea, Spain and Colombia) cluster in different clades (II, III and IV, respectively), so we argue that our study includes representative isolates from different clades. This suggests that the phenomena shown in this article are not restricted to an specific *C. auris* genotype. However, the same study demonstrated that several clades can also be isolated from the same country. For this reason, we believe that our work should be further extended and include representative isolates from all the five different clades that have been defined within this species [11,12,15,16].

PG occurs more frequently in the presence of echinocandins, especially CPF [36]. *Candida tropicalis* is the species where PG is most frequently observed [36,56]. Studies show that this phenomenon develops in a significant percentage of *C. auris* isolates from different geographical areas [29,57], while in *C. albicans* it occurs in about 6% of strains [36]. This is in agreement with our results, suggesting that it is not an isolated phenomenon for certain strains, but rather a characteristic phenotype of this species.

To date, *Candida* resistance to echinocandins is mainly caused by mutations in the *FKS* genes [57,58]. In our study, all *C. auris* isolates analyzed showed the same sequence in HS and corresponded to the sequence present in the reference genomes. By comparing the sequence of *C. auris* HS with those of other species, two substitutions in *FKS2* HS1 have been identified: F635Y and R641K. Mutations in these positions have been associated with the acquisition of resistance in *Candida* [58,59]. In particular, the change F635Y has been described for *C. glabrata* strains with reduced susceptibility to echinocandins [51]. However, its association with reduced susceptibility of *C. auris* is unknown. The susceptibility profile indicates that the growth of *C. auris* at different CPF concentrations was dissimilar to that observed for *C. albicans* strains that were fully resistant due to mutations in the HS regions. Our comparison of the expression of the *FKS* genes suggests that for *C. auris*, the main β-1,3-glucan synthase activity is encoded by *FKS1*, which contains the consensus HS sequences and is predicted to be fully susceptible to echinocandins. For this reason, we argue that the F635Y substitution in HS1 from *FKS2* plays a minor role in susceptibility to these antifungals. However, it is important to consider that the presence of this substitution and absence of consensus HS2 sequence in *C. auris FKS2* suggests that the affinity of echinocandins to this isoenzyme is significantly lower. As a consequence, any change in the balance of *FKS1/FKS2* gene expression, which results in a higher contribution of Fks2p in the β-1,3-glucan synthase activity, might translate in a resistant phenotype. In this regard, we found that after CPF treatment, expression of *FKS2* increased around 2-fold, a change not observed in *FKS1* expression, suggesting a compensatory mechanism to avoid the inhibitory effect of the antifungal. For *S. cerevisiae, FKS1* is the main gene expressed in glucose and *FKS2* is induced during starvation and in response to pheromones [60]. Additionally, and in agreement with our findings, *FKS2* is also increased in mutants that have a higher release of β-1,3-glucan from the cell wall [61], suggesting that it is regulated to compensate decreases in the amount of this polysaccharide in the cell wall.

The decrease in susceptibility observed in these isolates at high CPF concentrations could more accurately be described as drug tolerance, as would be expected from adaptive cell physiology due to environmental stress. It has been described that *Candida* species can induce tolerance to different antifungal families, such as azoles and echinocandins [38]. In the case of CPF, tolerance mainly involves cell wall rearrangements, such as increase in chitin synthesis. However, growth at high CPF concentrations is still severely impaired as cells cannot filament and typically show the phenotype of enlarged and abnormal cells [33,52,62]. The adaptation mechanisms to high CPF concentrations in *C. auris* have some similarities, but also differences compared with *C. albicans*. Although there was an increase in chitin content in *C. auris* in the presence of CPF, it was not as marked as observed in *C. albicans*. In addition, growth at high CPF concentrations of CalPG is associated with marked morphological changes not observed in *C. auris*. In general, our data led us to suggest two different hypotheses: Firstly, the cell wall structure in *C. auris* was not as dependant on β-1,3-glucans as in *C. albicans*. However, the fact that *C. auris* cells were inhibited by intermediate concentrations of CPF indicates that this cellular component is still important for the viability of the cells, suggesting that PG depends on adaptation mechanisms induced by high echinocandins concentrations. Secondly, an alternative hypothesis is that survival of *C. auris* in the presence of echinocandins requires induction of adaptive mechanisms, but this produces a different phenotype to those described in *C. albicans*. The best characterized adaptation mechanisms during PG involve overexpression of genes implicated in cell wall function, signal transduction and vacuole function [58]. Efflux pumps that expel antifungals from cells have been described as an important factor in resistance and biofilm formation [63]. However, in yeasts, these pumps seem to play a role mainly in resistance to azoles, as they have a very low affinity for echinocandins [58], but their role in *C. auris* remains unknown. In our work, we provide evidence of some of the mechanisms involved in *C. auris* adaptation to CPF. We have shown that the most expressed genes in this condition encode cell wall proteins, such as Pga31, Cwp1 and Pga30. *Candida albicans pga1^−/-^* mutants are hypersensitive to CPF and have a lower chitin level [64]. Furthermore, *PGA31* is repressed by glucose and induced by non-fermentative carbon sources, such as lactate [65]. Pga30 is another GPI-anchored protein, highly homologous to Pga31. Interestingly, Cwp1 encodes a cell wall mannoprotein anchored to β-1,3- and β-1,6-glucans which is present in *S. cerevisiae*, but does not have any homolog in *C. albicans*. Cwp1 localizes at the birth of the bud scar and increases expression in conditions where there is a release of β-1,3-glucan from the cell wall, as happens in *gas1* mutants [61]. *Candida auris* is evolutionary more closely related to *C. albicans* than to *S. cerevisiae* [18], but we identified 28 genes homologous in *C. auris* and *S. cerevisiae* but absent in *C. albicans*, suggesting that these genes come from a common yeast ancestor, but were lost in the evolution of *C. albicans*. The fact that one of the genes most expressed in *C. auris* in response to CPF was absent in *C. albicans* supports the hypothesis that the adaptation mechanisms of these two species to echinocandins are different.

When performing a functional analysis of the genes induced in *C. auris* by CPF we found different categories, some of them related to cell wall structure and synthesis, and others from membrane, extracellular, cytosol, nucleus, cellular bud, polarized growth, and endomembrane system. Even among these other families, many of the genes had functions related to cell wall regulation. In this way, around half of the upregulated genes had already been associated to the cell wall or regulated by CPF treatment. Among them, it was noteworthy that several chitin synthases were significantly upregulated, which is in agreement with the increase in this polysaccharide measured by fluorescence. Accordingly, we also observed increased the expression of genes involved in glucosamine metabolism, which is required for chitin synthesis [66]. Furthermore, genes that encode chitinases were repressed. Globally, the transcriptional regulation of chitin synthases and chitinases provides a validation of the RNAseq data. We also detected genes that encode MAPK, involved in the integrity of the cell wall and response to different types of stress. Bck1 (MAPKKK) and Mck1 (MAPK) are regulated by Pkc1 [67–70] and activated in response to cell wall stress, inducing, among other phenomena, chitin synthesis [43]. Absence of these kinases results in hyper-susceptibility to CPF [71–73]. Regarding Hog1, although it was originally described as a MAPK required for adaptation to osmotic stress, it has been shown that it can be activated in response to different types of stress [69]. Hog1 has been recently involved in CPF resistance and cell wall structure in *C. auris* [74]. Although the main activation of these proteins occurs by phosphorylation, the fact that there is also an increase in the expression of the corresponding genes supports that they play a key role in CPF adaptation.

Our gene expression analysis indicates that the cell wall reorganization required for adaptation to CPF also involves the reduction of some cell proteins, such as Iff4 and Rbt1 (involved in adhesion), Pga45 and Scw11 (glucanase). An interesting finding was that many genes repressed by CPF were involved in iron and zinc uptake (such as *PRA1, ZRT1, ZRT2, ZRT3, SIT1, FTR1, PGA7* and *HMX1*). We do not have a clear explanation about these findings, but our results warrant future studies to determine whether susceptibility to echinocandins in *C. auris* is influenced by metal homeostasis.

Finally, although it cannot be predicted whether *C. auris* will continue to be a cause of global concern in recent years, its emergence and social alarm created, not only at a hospital level, but also at a social level, justify future studies to characterize the mechanisms of resistance and virulence developed by this yeast.

## Supplementary Material

Supplemental Material

Supplemental Material

Supplemental Material

## Data Availability

RNAseq data (raw documents) are available at the Sequence Reads Archive (SRA, https://www.ncbi.nlm.nih.gov/sra) from the NCBI database (temporary Submission ID SUB8665675; BioProject ID PRJNA682422). The BioSample Accession numbers for the samples are SAMN16989163, SAMN16989164 and SAMN16989165 for the control samples (cells incubated in RPMI) and SAMN16989166, SAMN16989167 and SAMN16989168 for the three samples treated with CPF (8 µg/mL).
