# Supplementary material for: Adaptation of the emerging pathogenic yeast Candida auris to high caspofungin concentrations correlates with cell wall changes
Source: Virulence. 2021 Jun 28;12(1):1400–17. doi: 10.1080/21505594.2021.1927609 (PMC8244764; doi:10.1080/21505594.2021.1927609)
Supplement: Supplemental Material [file KVIR_A_1927609_SM8050.zip › Supplemental captions.docx]

**Supplemental tables**

**Supplemental table 1.** The document contains three sheets. The first sheet contains the complete list of genes in *C. auris* and their corresponding homologs in *C. albicans* and *S. cerevisiae*. For each gene, the change of gene expression (fold change) and p-value are shown. N/A, gene not present in *C. albicans* or *S. cerevisiae*. The second and third sheets contain the list of overexpressed genes (>2 fold, p<0.05) or repressed genes (<2 fold, p<0.05), respectively.

File can be downloaded from the following link:

<https://drive.google.com/file/d/1Y3Ju-DIfKm4O5n9ZnF3j3LqJnhiMgfkX/view?usp=sharing>

**Supplemental table 2.** Gene Onthology analysis of the overexpressed genes. The first sheet contains the main GO terms identified among the list of overexpressed genes (fold change >2, p value <0.05). In the second sheet, the list of genes was sorted to remove duplicates of the first sheet that were found in several categories. Furthermore, we merged the functional categories in three classes: Cell wall, chitin and RNA polymerase. Finally, the third sheet contains the list of overexpressed genes with the annotated functions in *C. auris*, *C. albicans* and *S. cerevisiae*. Not defined means that the gene is present in this species, but it has not been assigned a name. Not present means that the gene is not present in that species.

File can be downloaded from the following link:

<https://drive.google.com/file/d/103kx8ruJsxYKnl_1Ut3lerbKlcbLlO-f/view?usp=sharing>

**Supplemental table 3.** Functional classification of overexpressed genes in *C. auris* in the presence of CPF. Genes were associated to a cellular component of the cell using the GO Slim Mapper option (See Material and Methods). Then, we identified in each group genes with functions related to cell wall, chitin, caspofungin, GPI, glucan and integrity, and these genes were highlighted in color. Finally, these genes were assigned to included in the cell wall sheet.

File can be downloaded from the following link:

<https://drive.google.com/file/d/1BoACDQO8_AOyhhq-DILiYjYnghYShHAS/view?usp=sharing>

**Supplemental table 4.** Gene Onthology analysis of the repressed genes. Same as Supplemental table 2, but with the list of genes repressed in the presence of CPF (fold change <0.5, p value 0.05).

File can be downloaded from the following link:

<https://drive.google.com/file/d/1c0QSGN2g8hCwFBHw7L2-JOQXZRpDfVpn/view?usp=sharing>

**Supplemental figures**

**Supplemental Figure 1.** Characterization of the growth of *C. auris* isolates from different origins in the presence of micafungin (0.004-2 µg/mL). *In vitro* susceptibility profile for the seven isolates of *C. auris*. The growth percentage represents the percentage of OD measured at 530 nm.

**Supplemental figure 2.** Characterization of the growth of *C. albicans* strains in the presence of anidulafungin (0.008-4 µg/mL).

**Supplemental figure 3.** Image showing a representative trailing effect of tolerance of *C. auris* to micafungin. Antifungal susceptibility plates with micafungin were prepared as described in Material and Methods, and inoculated with the control strains (*C. krusei* ATCC 6258 and *C. parapsilosis* ATCC 22019) and two different *C. auris* isolates (CL-10836 and CL-10958). Plates were incubated at 35 ^o^C for 24 h and images were taken with a Nikon digital camera with a macro lens.

**Supplemental Videos**

**Supplemental video 1:** Real time visualization of growth of *C. albicans* CL-10432 in RPMI (Left video), or in the presence of caspofungin 0.5 mg/L (central video) or 8 mg/L (right video).

Video can be downloaded from the following link

<https://drive.google.com/file/d/1rm_apGPFjUb341nl7SkRWeLvDZ5bg6os/view?usp=sharing>

**Supplemental video 1:** Real time visualization of growth of *C. auris* CL-10836 in RPMI (Left video), or in the presence of caspofungin 0.5 mg/L (central video) or 8 mg/L (right video).

Video can be downloaded from the following link

<https://drive.google.com/file/d/1-sKCUOdRvOfErZkwLon2I8ok1kw69AYa/view?usp=sharing>
