# Supplementary figures and images for: Adaptation of the emerging pathogenic yeast Candida auris to high caspofungin concentrations correlates with cell wall changes
Source: Virulence. 2021 Jun 28;12(1):1400–17. doi: 10.1080/21505594.2021.1927609 (PMC8244764; doi:10.1080/21505594.2021.1927609)

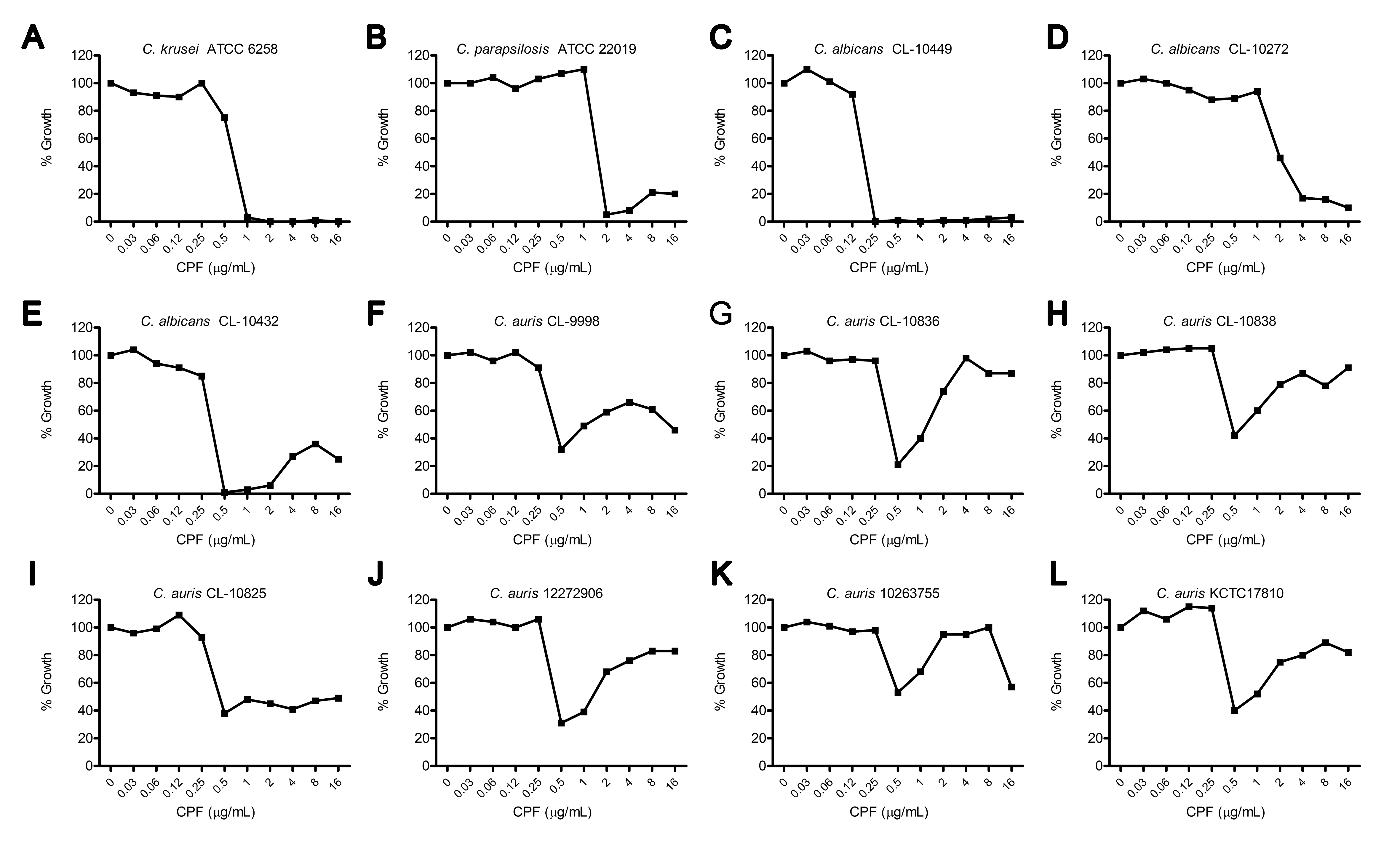

Supplement: Supplemental Material [file KVIR_A_1927609_SM8050.zip › Supplemental figure 1.tif]

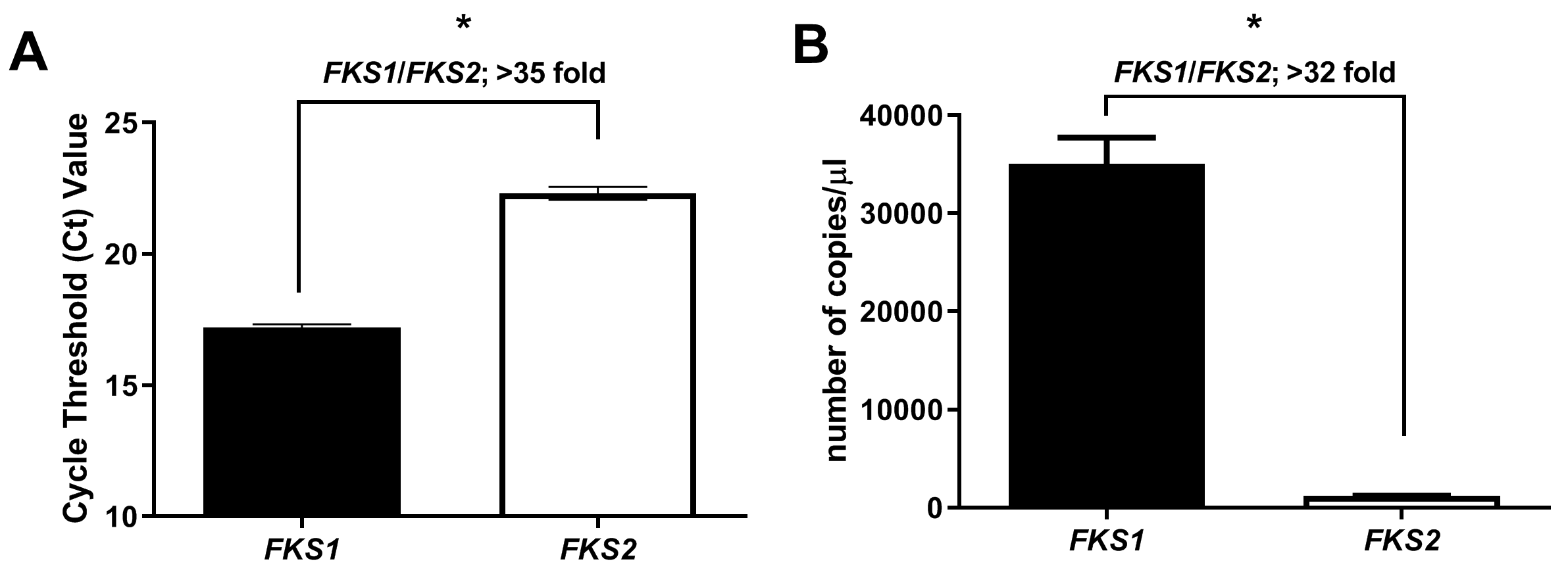

Supplement: Supplemental Material [file KVIR_A_1927609_SM8050.zip › Supplemental figure 2.tif]

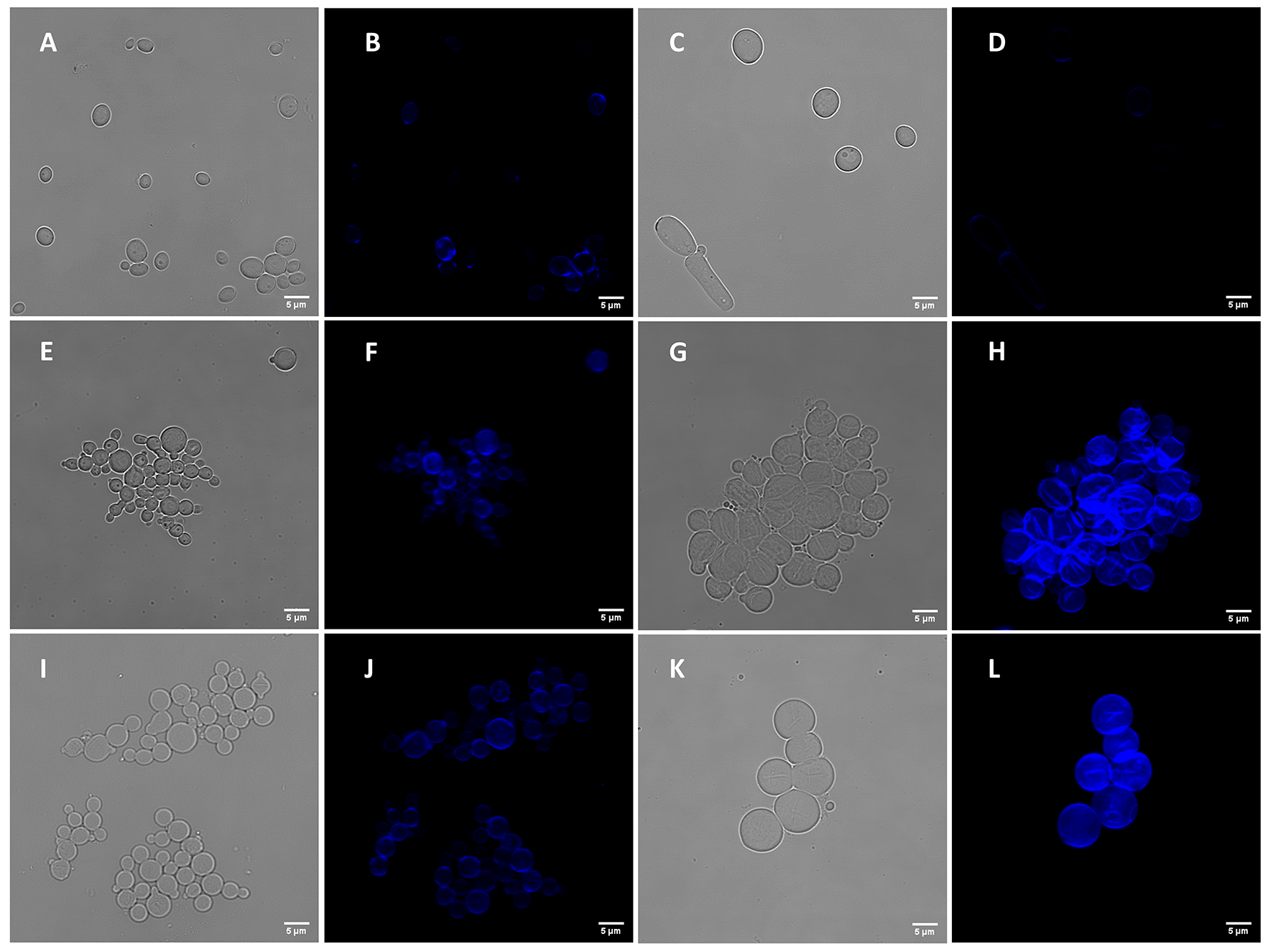

Supplement: Supplemental Material [file KVIR_A_1927609_SM8050.zip › Supplemental figure 3.tif]
